# Urban greenspace as a climate change adaptation strategy for subtropical Asian cities: A comparative study across cities in three countries

**DOI:** 10.1016/j.gloenvcha.2021.102248

**Published:** 2021-05

**Authors:** Leslie Mabon, Wan-Yu Shih

**Affiliations:** a Scottish Association for Marine Science, Oban PA37 1QA Scotland, United Kingdom; b Department of Urban Planning and Disaster Management, Ming-Chuan University, Taiwan

**Keywords:** Climate change adaptation, Environmental policy, Greenspace, Nature-based solutions, Urban planning

## Abstract

Interest in nature-based approaches for climate change adaptation in cities is growing. Whilst there is a growing field of scholarship in a European and North America setting, research on the policy and governance of urban greenspace for climate adaptation in subtropical Asia is limited. Given the different development patterns, environmental characteristics and governance arrangements in subtropical cities, plus their comparatively large population and high climate risk, this is a significant knowledge gap. In response, this paper evaluates competences – skill sets, capabilities, and supporting policy and legislation – to enact adaptation through greenspace across different governance contexts; and assesses how international rhetoric on nature-based adaptation becomes localised to subtropical Asian city settings. We conduct interviews with stakeholders, plus review of relevant policy and city-specific research, for three cities with different governance and development contexts: Hanoi (Vietnam); Taipei (Taiwan); and Fukuoka (Japan). Across all three cases, we find that institutional structures and processes for connecting different remits and knowledge systems are a bigger challenge than a lack of appropriate policy or individuals with the required technical knowledge. However, opportunities for civil society participation and consideration of justice issues vary between the cities according to the socio-political context. These findings illustrate the value of individuals and organisations able to work across institutional boundaries in linking greenspace and adaptation agendas for subtropical Asian cities; and the importance of competence in collaboration with developers and civil society so that the rapid development or regeneration seen in subtropical Asian contexts does not tend towards green climate gentrification. More broadly, our findings show that the diverse nature of subtropical Asian cities means the role of greenspace in climate adaptation is likely to be context-specific, and thus that caution must be exercised against uncritically importing best practices from exemplar cases elsewhere.

## Highlights

• Comparison of nature-based climate adaptation across subtropical Asian cities;
• Dense urban form and diverse governance structures in Asian cities compared to West;
• Competences as organising framework to assess policy and skills for adaptation;
• Capability to link government departments and sectors of society vital;
• Further research needed for normative competence under different governance forms.

## 1 Introduction

Amidst interest in cities as sites for climate resilience and sustainability action, recent years have seen significant research, policy and practice discussion on urban nature-based solutions as a response to contemporary environmental and social challenges. Yet scholarly engagement with the governance and policy aspects of nature-based approaches to urban climate adaptation outside of Europe and North America is limited (Escobedo et al., 2019). Conversely, Friend and Moench (2015) hold that the most dramatic processes of urbanisation are happening in Asia, in locations that are by nature hazardous, and where land use and consumption changes driven by investment have the potential to intensify risks from climate change. Tropical zone cities, especially in Asia, are argued to face heightened exposure to extreme events associated with climate change (e.g. Friend et al., 2014, Giridharan and Emmanuel, 2018). Moreover, subtropical Asian cities may be governed under a breadth of political systems – from authoritarian to new democracy through to more established democracies – that can influence the way in which urban nature is managed and to what effect (Dobbs et al., 2014, Han, 2017, Moser, 2020). Even in cities in subtropical Asia with more established climate adaptation approaches, density and urban development can put pressure on green spaces, to the detriment of less advantaged residents (e.g. Tan et al., 2016; Tan and Samsudin, 2017, Mabon, 2020). Accordingly, Friend and Moench (2015: 643) argue that “(a)t the heart of urbanization in Asia […] are challenges of governance and equity […] Issues of governance and equity link strongly to questions regarding how the urban future is shaped, for whose benefit and by whom.”

The specific governance question we address is thus: can we identify characteristics or skill sets that enable effective and equitable climate adaptation via greenspace within dense subtropical Asian cities, where there may be significant competition for land, high exposure to hazards and subtropical ecosystems, and potentially very different governance arrangements between city contexts? This question is significant for nature-based adaptation in cities, given Keeler et al (2019: 35) note that “conclusions drawn from [already well-studied locations] are of limited utility in the regions of the world that are projected to experience the greatest and most rapid urban growth.” There is growing scholarly interest in policy mobilities and the localisation of knowledge for green resilient cities (Acuto and Leffel, 2020, Affolderbach et al., 2019, Chang et al., 2020), and continued enthusiasm for globalised city-to-city networking at the science-policy interface (ICLEI-CBC, 2017, Bai et al., 2020). Yet within scholarship on urban climate change and sustainability, there is concern that cities outside of global- or ‘exemplar’ status are sidelined or missing in prominent research, policy and practice discussions, despite being the locations in which most people will experience climate change (Castán Broto, 2020, Ruszczyk and Price, 2019). It is hence important to make sense of how understandings and practices of nature-based adaptation may be ‘localised’ in subtropical Asian settings to reflect social, political, cultural and environmental contexts. Indeed, looking to locations that are somewhat ‘off the map’ of nature-based adaptation expertise may yield insights which can help to address equity and justice concerns seen in Western locations (Shi, 2020).

Nonetheless, enquiry into greenspace and adaptation in subtropical Asian cities requires understanding of the specific urban development characteristics of such locations. Whilst compact cities have been widely accepted as a sustainable urban development form to tackle problems arising from sprawl (Westerink et al., 2013), many populous Asian cities have embedded the development characteristics of high density and mixed land use. The speed of economic growth and rapid urbanisation over a short timespan faced by tropical zone Asian cities can produce distinct challenges. To some extent, this benefits citizens through greater accessibility to public services and infrastructure so as to reduce the need of transportation. However, Asian cities often struggle with problems related to density, such as low levels of greenspace per capita, environmental pollution, and intense urban heat island effects (Haaland and van den Bosch, 2015, Shih and Mabon, 2020). Protecting and reintroducing greenspaces and ecosystem services into compactly developed cities are increasingly regarded as key for quality densification, which improves the health and resilience of urban socio-environmental systems (Tappert et al., 2018, Shih and Mabon, 2020). Yet, the loss of greenspaces is generally more prominent in Asian cities, especially in developing countries (Haaland and van Den Bosch, 2015). The types of challenges regarding protection and/or creation of urban greenspaces varies with the development states of cities. Cities in low- or middle-income Asian countries often experience significant loss and fragmentation of natural areas, including greenspaces and water bodies, due to rapid rural to urban migration, which increases demand for new housing, and the prioritisation of economic profits from construction, (e.g. Shibayama, 2009, Pham and Nakagoshi, 2008). Conversely, for cities with slow population growth or even decline, urban greenspaces might be characterised by dynamic loss and gain depending on greening policies and strategies for infill development, regeneration, and/or expansion at different sites across the city (Haaland and van den Bosch, 2015, Shih and Mabon, 2020).

This paper responds by considering competences for climate adaptation via urban greenspace in three subtropical Asian cities at different urban development stages – Fukuoka in Japan; Hanoi in Vietnam; and Taipei in Taiwan. The three cities also have different governance arrangements – Hanoi as authoritarian but with a liberalised economy and an increasingly international outlook; Taipei as a new and flourishing democracy; and Japan as an established yet ‘policy-driven’ democracy. The three cases thus provide fertile ground for assessing the messiness and complexity of urban climate change responses (Castán Broto, 2020) in the kinds of cities where such interventions are likely to be most needed.

## 2 An evaluative framework of competences

We work with the idea of competences as a way to understand what is required to enact climate adaptation via greenspace across a breadth of subtropical Asian city contexts. Renn et al (2013: 58) define competence in making decisions in society as the ability to construct “the most valid understandings and agreements possible given what is reasonably knowable at the time”. Holtz et al. (2018) argue that competences encompass both formal powers to set laws, policies and plans (Jordan, 1999); and a broader set of skills and capabilities which allow individuals and institutions to tackle complex sustainability issues (Wiek et al., 2011). We are primarily interested in the competences held by local government departments and the individuals within them, given the importance of local-level plans, policies and legislation in setting a vision and initiating strategic urban greening benefits (Gradinaru and Hersperger, 2019) and hence realising the kind of strategic, planned action that is required for climate change adaptation via urban greening (Tan et al., 2013). This is especially so in tropical contexts, where top-down modes of governance may be more prevalent (Dobbs et al., 2014). However, where appropriate, we also refer to competences held by individuals and institutions in the wider governance system who may have a role to play in turning urban greening policy and rhetoric into reality, for instance civil society organisations, private sector developers, and communities (Nemoto and Biazoti, 2017).

Competence-based approaches emphasise *application* of knowledge across different systems (Jacobsson and Karltorp, 2012, Kerry et al., 2012); and ability to enact interventions and conduct change processes (Perez Salgado et al., 2018). Climate change adaptation and resilience-building via urban greening is a complex issue requiring social, ecological and technological aspects to be considered together (Keeler et al., 2019), yet one where evidence of successful interventions across different city contexts is still emerging despite significant high-level policy rhetoric (Dorst et al., 2019, Douglas et al., 2021, Garmendia et al., 2016). Characterising the competences required to facilitate climate change adaptation via greenspace in subtropical Asian cities therefore offers insight into how to turn rhetoric on nature-based adaptation into tangible outcomes to reduce climate risk and build resilience. Furthermore, Wiek et al. (2011) acknowledge a need remains to justify why certain competences are necessary based on empirical evidence; and Perez Salgado et al. (2018) call for more exploration of intervention competences across different social and cultural settings. Our paper thus uses empirical enquiry to make the case for why these competences are necessary to facilitate adaptation through urban greenspace in subtropical Asian cities, and illustrates what these competences might look like in practice.

When we evaluate competences in climate adaptation via greenspace, we are therefore assessing both formal policies and legislation to enable nature-based adaptation, and also the wider capabilities and skill sets held by individuals and institutions which allow adaptation action to progress in a manner appropriate to the city context. This focus on underlying capabilities and skill sets, rather than purely on specific policies or technologies, is intended to draw out wider learnings across different city contexts and political systems. By assessing the relative strengths and weaknesses of activities in different local contexts, we focus on understanding skill sets for progressing adaptation via greenspace in subtropical Asian cities, rather than claiming one city is ‘better’ than another or looking for best practice examples. Moreover, we acknowledge there are competing understandings of terms such as ‘resilience’ and ‘adaptation’. Whilst a full interrogation of these terms is beyond the scope of the paper, Table 1 sets out how we understand key terms and the relation between them.

**Table 1:** Key terms as understood in paper.

Term	Definition	Indicative references
Adaptation/climate change adaptation	Activities and strategies to reduce risk and vulnerability to climate changes, in a way that moderates harm to natural and social systems and exploits opportunities.	Hughes (2015)
Greenspace	“vegetated urban land that is public or semi-private […] such as parks, sports fields, cemeteries, vegetated areas of street and road corridors […], natural and built corridors adjacent to waterways and wetlands, and external areas to public buildings” (Boulton et al., 2018: 84)	Boulton et al (2018)
Nature-based solutions	The “maintenance, enhancement, and restoration of biodiversity and ecosystems as a means to address multiple concerns simultaneously” (Kabisch et al., 2016: 1) to bring environmental, economic and societal benefits towards resilience. Greenspaces may be considered part of nature-based approaches if maintained to deliver benefits in this way.	Kabisch et al., 2016, Keeler et al., 2019
Resilience	Ability to “maintain or rapidly return to desired functions in the face of a disturbance, to adapt to change, and to quickly transform systems that limit current or future adaptive capacity.” (Meerow et al., 2016: 39). Enhancing resilience is a core outcome of climate change adaptation activities and strategies.	Meerow et al (2016)

We derive our analytical framework from the climate adaptation competences approach developed by Mabon (2018), which itself is developed from the sustainability competences framework of Wiek et al (2011) that has been applied across a breadth of sustainability studies (Ploum et al., 2018, Redman et al., 2020). The five competence categories of this framework provide an organising structure for a much wider suite of competences identified in existing environmental literature. *Setting goals, targets and outcomes through policy and leadership* reflects competence in efficient use of space and the use of spatial planning (Holtz et al., 2018, Kerry et al., 2012); providing guidance by laying out a vision (Holtz et al., 2018, MacDonald et al., 2020); and linking action across scales (Solís-Espallargas & Morón-Monge, 2020). *Defining, developing and realising pathways* brings together ability to ‘get things done’ and knowing how to act (Wiek et al., 2011, Kerry et al., 2012, MacDonald et al., 2020); experimentation and social learning (Holtz et al., 2018); political-strategic thinking to span multiple perspectives linking government and private sector actors (Perez Salgado et al., 2018); and mobilisation and use of resources (Holtz et al., 2018, Solís-Espallargas and Morón-Monge, 2020). *Availability, synthesis and use of knowledge* brings together linking knowledge systems (Jacobsson & Karltorp, 2012); linking lived experience to scientific knowledge (Perez Salgado et al., 2018); and possession of good knowledge as well as knowing how/when to call on expertise (Kerry et al., 2012, MacDonald et al., 2020). *Civil society collaboration* (which we understand as collaboration with public and civil society actors rather than governments or developers) requires steering stakeholder diversity into common and shared positions (Perez Salgado et al., 2018); supporting network-building (Holtz et al., 2018); and understanding, comparing and critically evaluating different positions (MacDonald et al., 2020, Wiek et al., 2011). Lastly, *ethical and normative competence* considers how social-ecological systems *ought* to be developed (Wiek et al., 2011) and the practical application of ethical principles (Perez Salgado et al., 2018, Solís-Espallargas and Morón-Monge, 2020). Table 2 illustrates what these competence areas involve, and why they may be necessary, for adaptation and greenspace.

**Table 2:** Competences and sub-competences for adaptation via greenspace, and justification for inclusion.

Competence area	Fit with underpinning literature	Sub-area for adaptation in greenspace	Justification and indicative references
1. Goals, targets and outcomes through policy and leadership	Spatial planning and efficient use of space (Kerry et al., 2012, Holtz et al., 2018)	Policies, legislation and plans for (a) greenspace and (b) adaptation	Policies and plans fundamental for setting out visions and how these will be realised across space, especially given move towards thinking of greenspace in terms of city-wide network delivering functions (Gradinaru and Hersperger, 2019)
	Linking action across scales (Solís-Espallargas & Morón-Monge, 2020)	Mechanisms/ effectiveness of integrating across sectors	Both ecosystem services and adaptation cut across sectors, hence need to mainstream across different areas of urban governance to realise fuller potential (Dorst et al., 2019, Wamsler et al., 2014)
		Mechanisms/ effectiveness of integrating across different levels, from national to local	Municipal-level greening actions may be informed by national/regional-level legislation, and are contingent on local on-site actors for implementation (Kabisch, 2015)
	Providing guidance by laying out a vision (Holtz et al., 2018, MacDonald et al., 2020)	Presence of leadership and champions	Leaders/champions vital to both set a vision for city-wide greening and put it into practice, given relatively novel concept of resilience through urban greening (Newman, 2014)
2. Defining, developing and realising pathways from the present towards envisioned outcomes	Political-strategic thinking to span multiple perspectives (Perez Salgado et al., 2018; MacDonald et al., 2020)	Rationales/justifications for greenspace provision	Political and societal vision can inform planning approaches and the purpose/configuration of green spaces, reflecting state views of how nature ought to be governed (Han, 2017, Badiu et al., 2019)
		Linking of greenspace and adaptation with socio-economic development	Connection to socio-economic development goals – especially poverty alleviation in developing countries – can transcend idea of greenspace preservation being opposed to development (Shih & Mabon, 2017)
	Experimentation and social learning (Holtz et al., 2018); understanding broader global context (Kerry et al., 2012)	Opportunities for innovation, experimentation and learning	As nature-based adaptation a new approach, experimentation valuable to understand which tools and programmes are most effective locally (Frantzeskaki, 2019)
		Participation in knowledge-sharing within city and internationally	Policy mobilities important in urban greening to share knowledge and compete for leadership (Affolderbach et al., 2019); may enable ‘Global North’ to learn from ‘Global South’ for nature-based adaptation (Shi, 2020)
	Mobilisation of resources (Holtz et al., 2018, Solís-Espallargas and Morón-Monge, 2020)	Ability to access long-term and self-sustaining funding	Nature-based approaches need to compete with other areas for municipal funding – especially traditional grey infrastructure / technological solutions (Keeler et al., 2019)
3. Availability, synthesis and use of knowledge	Linking knowledge systems to understand complex problems (Jacobsson & Karltorp, 2012)	Comprehensive environmental data to support evidence-based decision-making, relating to (a) climate and (b) greenspace	Knowledge and frameworks can improve understanding of cities as complex systems, and role of greenspace and biodiversity within urban ecosystem (Tan et al., 2013)
		Capabilities of policy-makers and stakeholders involved in reaching and implementing decisions	Attaining resilience through nature-based approaches requires capability at local level to integrate knowledge systems for planning and management (Frantzeskaki et al., 2016)
	Knowing when and how to call on expertise (Kerry et al., 2012, MacDonald et al., 2020)	Decision-support tools to help non-technical officials understand greenspace and adaptation	Data can often be complex for urban greenspace and climate issues and/or require new ways of thinking about greenspace function, may require knowledge brokers to interpret/translate (Brink et al., 2016, Mabon and Shih, 2018)
	Connecting lived experience to scientific knowledge (Perez Salgado et al., 2018)	Processes to integrate different kinds of expertise in decision-making	Whilst there is strong natural science data, attention to local knowledge, and to humanities and social science, allows more nuanced understandings of resilience to emerge (Borie et al., 2019, Brink et al., 2016)
4. Civil society collaboration	Understand, compare and critically evaluate different positions (Wiek et al., 2011, MacDonald et al., 2020); network-building (Holtz et al., 2018)	Approaches to support cooperation with and participation from civil society and communities	Governance of nature-based adaptation can cut across different sectors with different priorities – more attention to political processes in decision-making and questions of inclusion could help to deal with trade-offs (Dorst et al., 2019)
		Channels for public participation in decision-making	Engagement with citizens important to build understanding of and support for greenspace interventions for adaptation, which may be new and unfamiliar (Byrne et al., 2015)
	Steering stakeholder diversity (Perez Salgado et al., 2018)	Effectiveness of participatory processes on outcomes for greenspace and adaptation	Meaningful and effective participation can create positive relationship with management and design, and in turn enhance ecosystem services from greenspace (Dennis & James, 2016)
5. Ethical and justice issues	Vision of how socio-ecological systems ought to be developed (Wiek et al., 2011)	Equitable benefit from key adaptation assets (e.g. ecosystem services) provided by greenspaces	Extant research shows pattern of unequal exposure to environmental risks, and unequal exposure to benefits of urban nature, across cities – low-income and ethnic minority communities often disadvantaged (Keeler et al., 2019)
	Application of ethical principles in practice (Perez Salgado et al., 2018; Solís-Espallargas & Morón-Monge, 2020)	Processes to understand differences in vulnerability across society and space	Although research into links between vulnerability and nature-based adaptation emerging, little evidence explicit to subtropical cities where not only ecosystems but also socio-cultural relations to urban nature may differ (Mabon, 2020)
		Explicit consideration of justice issues in municipal greenspace planning for adaptation	Greenspace polices in the name of adaptation may disproportionately accrue to privileged groups, or lead to inequitable outcomes – hence need for explicit consideration of justice issues at planning stage (Haase et al., 2017, Shokry et al., 2020)
		Measures to reduce inequalities and/or benefit the most vulnerable at climate/greenspace interface	View of greenspace as inherently ‘good’ risks obscuring inequalities, hence need to actively prioritize outcomes for vulnerable groups disadvantaged as a result of historical and contextual factors (Anguelovski et al., 2020, Hoffman et al., 2020, Nyelele and Kroll, 2020)

These competences are especially important given the complexities of climate adaptation via greenspace in comparison to more conventional greenspace planning. Climate regulation functions from greenspaces, such as microclimate regulation and runoff management, are related to topographical characteristics and configuration of green infrastructure (e.g. Shih, 2017). These functions tend to decay with distance and are more likely to have effects locally (Hough, 2004, Kabisch and Haase, 2014, Shih and Mabon, 2020). However, conventional greenspace standards in urban planning focusing on availability and accessibility for recreation are unlikely to address these spatial characteristics of ecosystem services. A key reason is that climate adaptive planning via greenspaces requires integration of multiple technical expertises, such as climatology, hydrology, ecology, and epidemiology, into land use planning systems which are not necessarily designed to incorporate diverse knowledge systems at the local level (Davies and Lafortezza, 2017). Furthermore many natural spaces, such as wetlands, woodlands, rivers, and ponds, are managed separately by various authorities. The lack of mechanisms for holistically and systematically governing, planning and managing natural elements across a city-region for climate regulation functions has been a major obstacle for enhancing the resilience of a city to climate-related stresses (Shih and Mabon, 2018a, Shih and Mabon, 2018b).

## 3 Background to cities

The competences in Section 2 are evaluated through application to Fukuoka; Hanoi; and Taipei. Table 3 summarises the main characteristics of these cities. Their urban populations range from approximately 2.5 million to 8.6 million; and the cities are rated from Alpha (Taipei) through to Beta (Hanoi) and Sufficiency (Fukuoka) on the Globalisation and World Cities (GaWC) 2018 rankings (Globalisation and World Cities Research Network, 2018). The three cases also represent different governance systems. Hanoi represents an authoritarian government, albeit one with a liberalising market economy and increasing international investment and knowledge-sharing on climate change issues (Leducq and Scarwell, 2020). Taipei represents a relatively new democracy following the end of Marital Law in 1987, with a vibrant civil society movement and significant enthusiasm at city and national government levels for new forms of participatory democracy (Fan, 2021). Fukuoka, meanwhile, represents a longer-established democracy, but one in which opportunities for citizen and civil society actors to influence policy and planning decisions may be limited within more top-down and technocratic greenspace planning processes (Mabon et al., 2019a, Mabon et al., 2019b). The three cities hence cover a breadth of different governance forms and socio-economic development stages despite similar climate characteristics, and thus allow us to explore the question of whether there are common skill sets that can help to resolve the governance challenges that extant literature (e.g. Friend and Moench, 2015, Moser, 2020) see as key to adaptation and resilience in Asian cities. Moreover, the three cases may yield valuable insights into how subtropical Asian cities can respond to multiple adaptation challenges through greenspace, but perhaps have not received the international research or policy-practice attention of other Asian cities in the Tropics (e.g Singapore, Shenzhen) which have been evaluated positively for their vision, leadership and evidence-driven greenspace policy (e.g. Biophilic Cities Network, n.d.; ICLEI-CBC, 2017). Looking to three cities spanning different governance systems and development stages, which are all starting to address adaptation challenges via urban greening, can thus contribute to the emerging conceptual challenge of understanding how international rhetoric on nature-based adaptation becomes localised into different contexts which may be ‘off the map’ of prominent work to date at the science-policy interface (Chang et al., 2020, Shi, 2020).

**Table 3:** characteristics of case study cities.

City	Fukuoka	Hanoi	Taipei
Population (core city area)	1,588,924 (2015)	3,642,131 (2014)	2,674,063 (2018)
Population (wider urban area)	2,565,501 (2015)	7,781,631 (2014)	8,605,000 (2018)
Characteristics of city growth in the past 5 years (core city area)	Regeneration in city centre - especially ‘Tenjin Big Bang’ core area designated by city government - and expansion to west	Rapid growing city: infill development, and rapid urban expansion	Out-migration city: infill development, regeneration, and new development in the urban fringe
Greenspace loss or gain (core city area)	Increase in area of formal greenspaces through incorporation of informal greenspace into new parks, but decrease in greenery across city overall, especially with development in west of city	Radical loss and fragmented	Loss in new development area, but gain through urban regeneration programmes
Greenspace per capita	Official parks and greenspaces: ranging from 2.5 m^2^ per person to 17.52 m^2^ per person in seven districts (Mabon et al., 2019a)	Official parks and greenspaces: ranging from 0.25 m^2^ per person to 2.58 m^2^ per person in ten central districts (Nguyen, 2018)	Official parks and greenspaces: ranging from 2.11 m^2^ per person to 10.95 m^2^ per person in thirteen districts (DBAS, 2020)
Climate	Humid subtropical (Cfa)	Humid subtropical (Cfw)	Humid subtropical (Cfa)
Government	Fukuoka City Government	Hanoi People’s Committee	Taipei City Government
Democracy Index (2019)	7.99 Flawed democracy	3.08 Authoritarian	7.73 Flawed democracy
National Human Development Index	Rank 19 (0.909) (2018)	Rank 116 (0.694) (2018)	Rank 21 equivalent (0.907) (2018)
Globalization and World Cities Research Network Classification (2018)	Sufficiency	Beta+	Alpha
Main climate risks identified by city in city climate adaptation plans	Flooding/heavy rainfall; pressure on water resources; heat risk; biodiversity loss; effect on agricultural produce (Fukuoka City, 2016)	Flooding; drought; pressure on water resources (Nguyen Phuong Nam et al., 2015)	Flooding, landslide, drought, extreme temperature, sea level rising (Huang et al., 2012)
Participation in international climate/ sustainability networks	Host city for UN Habitat regional office	C40 Cities; collaboration with ICLEI South East Asia Secretariat as model city of Ambitious City Promises in Vietnam	Global Covenant of Mayors for Climate & Energy; ICLEI; Future Earth

For analytical consistency, we focus on the policies and plans set by the government of the core city area (i.e. Fukuoka City Government; Hanoi People’s Committee; Taipei City Government); and draw in issues and examples from other levels or areas of government (e.g. national governments, regional governments, adjacent municipal governments) where relevant. Indeed, reflection on the linkage between different areas and different levels of government – and the challenges and slippages this may entail – forms part of both the Findings (Section 5) and Discussion (Section 6).

## 4 Method

We use a combined approach of in-depth interviews and content analysis of core policy plans and documentation relating to greenspace planning and/or climate change adaptation to evaluate competence areas in the case study cities. Analysis of high-level overviews of strategies and other textual materials produced by cities has been used by other recent research (e.g. Castán Broto et al., 2019, Meerow et al., 2019) to clarify issues relating to adaptation and resilience across a broad range of geographical and development status contexts. In our study too, we analyse the content of relevant city plans and policies (e.g. climate adaptation strategies, urban plans, greenspace plans) and scholarly texts produced by local researchers. We do so to understand how the three cities are considering greenspace planning as an adaptation strategy to climate change, and to gain insight into how the competences outlined in Section 2 may manifest themselves in the greenspace and adaptation planning activities of the cities.

However, in this study we focus on a smaller number of cities in-depth, to build richer contextual understanding of the opportunities, practices and challenges faced in adaptation via greenspace in each case. Norton (2008) warns that content analysis of plans and policies may overstate the quality of action being undertaken in a locale, if one reads plans only for the presence of certain features and not for the way in which these are discussed. This need to go beyond what is stated in documentation is pertinent given our interest in identifying and assessing the competences driving each city’s adaptation and greenspace planning and policy actions. Moreover, bearing in mind Borie et al. (2019) and the potential for multiple narratives of resilience to exist underneath apparent consensus, we aim to encompass more critical or nuanced perspectives on the rhetoric of ‘official’ narratives of resilience and adaptation produced by cities themselves. Accordingly, in-depth interviews (21 in total) were conducted in all three cities to understand the skill sets involved in turning policy into practice, and also to obtain more critical perspectives on the challenges faced. Sampling was focused on those with specific knowledge of the local greenspace and adaptation context, and aimed to cover both those tasked with setting and influencing policy (e.g. local government officials from urban planning, greenspace or environmental sectors; consultants) and also those able to clarify challenges and limitations from a position of expertise (e.g. academics, NGOs). Table 4 shows the full list of interviewees. The aim was to obtain a small yet focused sample, allowing us to explore in depth the views of those with rich knowledge of the context in each locality and hence gain insight into a complex topic requiring significant technical and scientific expertise. This approach allowed us to build a fuller understanding of the greenspace and adaptation landscape in each city to supplement material obtained from documentation. Interviews followed a semi-structured format, with each covering the competence areas outlined in Section 2 plus additional questions specific to the local context.

**Table 4:** overview of interviewees.

	Interviewee	Sector
Fukuoka	City government greenspace planning division	Local government
	City government environment division	Local government
	Prefectural government environment division	Regional government
	Regional environmental NGO	Civil society
	Academic involved in municipal climate plan expert committee	Academia/research
	Environmental research institute	Academia/research
Hanoi	Economic forecasting division, Ministry of Planning and Investment	National government
	Academic with expertise in greenspace planning	Academia/research
	Academic with expertise in greenspace and climate adaptation	Academia/research
	Climate change researcher at government research institute	National government/ Academia/research
	Academia with expertise in urban planning	Academia/research
	International development agency	Civil society
	Urban planning consultant	Private sector
	International organisation for urban sustainability	Civil society
Taipei	Urban planning consultant	Private sector
	Greenspace planning consultant	Private sector
	Landscape architecture and planning consultancy	Private sector
	Local government land administration division	Local government
	Local government urban development division	Local government
	Academic with expertise in urban planning	Academia
	Academic with expertise in urban planning/Urban Planning Committee member	Academia

Both policy documentation and interviews were reviewed and coded for places where the different competences laid out in Section 2 were mentioned. For each city, evidence – either from policies/plans or from the interviews – was noted alongside each competence sub-area.

## 5 Findings

**Fig. 1:**
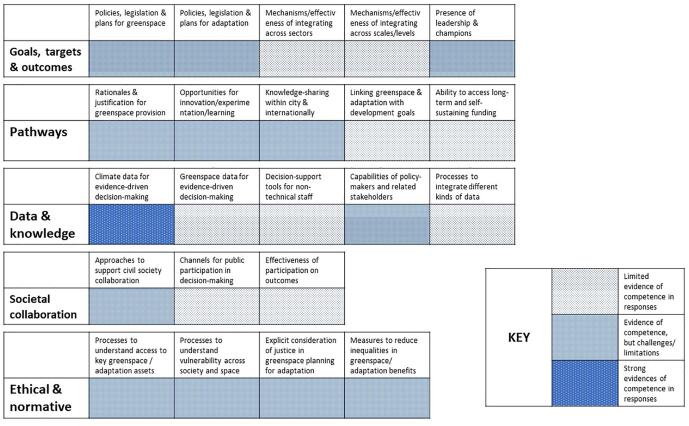
Hanoi competence summary

**Fig. 2:**
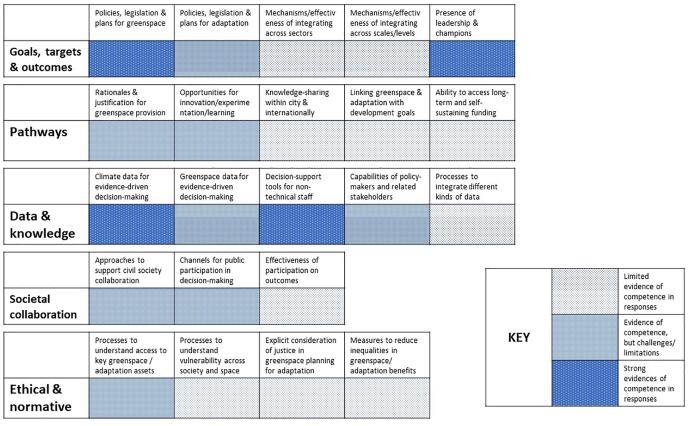
Fukuoka competence summary

**Fig. 3:**
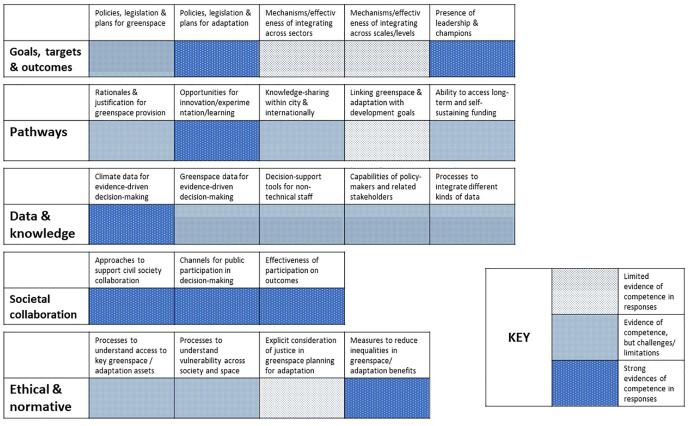
Taipei competence summary

This section summarises the findings for each city, drawing out areas of commonality and difference. For each city, a summary figure showing how respondents assessed the areas of competence for their own city is included (see Fig. 1, Fig. 2, Fig. 3). These figures are intended as a visual aid to understand how respondents saw the *relative* strengths and weaknesses of competence in their own cities, and do not represent a city-to-city comparison. A full inventory of the documents and interview statements on which the findings are based is included as Supplementary Material.

### 5.1 Goals, targets and outcomes through policy and leadership

Reflecting competence as both legislative power and skill in setting a vision, all three cities have *policies, legislation and plans* which may form the basis for climate adaptation via greenspace, and some form of greenspace plan. Taipei and Fukuoka have specific city-wide greenspace plans, and Hanoi has a greenspace vision for the whole city in the 2030 Masterplan, with separate ward-level greenspace plans. Taipei has a specific climate adaptation plan, whereas Fukuoka has an adaptation section within its climate change plan. Hanoi People’s Committee has an overarching decision regarding climate change countermeasures with additional plans linked to climate adaptation.

Across all three cities, respondents referred to these visions for climate change adaptation and urban greening – or at least for building a society more resilient to climate changes – when discussing their actions. However, in all cases, respondents felt weaknesses in *mechanisms for integrating across sectors* and *mechanisms for integrating across scales* prevented plans being realised. In both Taipei and Fukuoka, the difficulty of linking adaptation and greenspace into overarching urban plans, which ultimately set what can be done across space, was raised. For Hanoi, the difficulty was more to do with negotiating a whole range of competing and sometimes contradictory plans for the urban environment produced by different sectors. Enforcement of adaptation and greenspace plans was raised for Taipei and Hanoi; and whilst Fukuoka did not seem to face enforcement issues, specific plans (e.g. Fukuoka’s vision to cool the city centre via air flow and strategic greening) still did not get support from budgets or building codes to realise. Respondents in all three cities also argued greenspace- and adaptation planning were not always well-connected. One notable difference, however, is that both Taipei and Fukuoka have longer experience of natural hazards which supports planning for flooding in particular; whereas Hanoi faces continuing prioritisation of mitigation actions over adaptation due to the more visible and pressing perception of issues such as low-carbon transportation.

Similarly, for integration across levels of government, in all three cases different city government departments report to different national ministries. For example, in Fukuoka the Green City Promotion Division reports to the Ministry of Land, Infrastructure, Transport and Tourism; whereas the Environment and Energy Division (responsible for climate change) reports to the Ministry of Environment. Respondents suggested these vertical integration issues lead to different local governmental departments working to differing remits, which in turn limits institutional competence in tackling problems like adaptation via greenspace that require cross-departmental collaboration. Political processes at the national level also influence priority (Taipei) or speed of decision-making and budgetary approval (Hanoi), which constrain actions that can be taken locally. Conversely, national-level orders can empower cities with legislative competence to act, such as Taiwan and Japan mandating local municipalities to produce adaptation plans. For example, the new regulations regarding ‘runoff distribution’ and ‘runoff control’ in the Water Act enacted in Taiwan since 2019 enforce all urban plans to adopt land use strategies (particularly greenspaces) for mitigating flood risk.

Under a challenging policy landscape, it is not surprising that *presence of leadership and champions* was argued in all cases to be necessary for setting a vision for adaptation through greenspace. The importance of support from the highest levels of local government in creating favourable conditions for adaptation and greenspace was clear – whether the Hanoi People’s Committee and in particular the Department of Natural Resources; Head of Land Administration Department of Taipei City; or Mayor of Fukuoka. Champions working at the science-policy interface (e.g. for heat mitigation in Fukuoka and flooding in Taipei) or the practice-policy interface (e.g. landscape consultants in Taipei) were also reported to be important in raising technical awareness of the adaptation potential of greenspace, and facilitating cross-sector dialogue. Taipei was perhaps the city where champions had achieved the most in both influencing a vision and subsequently driving it forwards, as seen in, for example, the Taipei Smart Ecological Communities and Taipei Garden City initiatives. What made these initiatives successful was arguably cross-departmental collaboration within discrete programmes, where motivated individuals and departments were able to facilitate face-to-face dialogue and overcome institutional silos.

Nevertheless, in Taipei at least, respondents acknowledged that whilst the competences of individual champions were important in making small practical gains, they were not a substitute for a wider programme of rigorous evidence-informed decision-making (i.e. institution-level competence) towards adaptation via greenspace. As we now discuss in more depth, there is also difficulty in all three cities in progressing a vision beyond piecemeal or flagship project-based greenspace implementation for adaptation, towards comprehensive, city-wide, longer-term planning and sustained actions.

### 5.2 Defining, developing and realising pathways from the present towards envisioned outcomes

Respondents and reviewed documentation indicate the respective city governments have different *rationales and justification for greenspace provision*, reflecting local social contexts and political priorities. Hanoi, for instance, emphasises the cultural significance of greenspace through allusion to the role of greenspace creation in post-war unification such as the Thong Nhat (Unification) Park (Pham et al., 2013); and the desire to develop Hanoi as a distinctly Vietnamese biophilic city via the *One Million Trees* initiative. In Taipei, parks and greenspace systems in the urban plan have traditionally emphasised the physical health and recreational benefits of greenspace; whereas city authorities in Fukuoka have focused on the contribution of greenspace to a ‘liveable environment’ encompassing recreation, amenity and aesthetic quality. Whilst none of these rationales are closely linked to climate adaptation, discourse in each city has shifted recently towards a more climate-focused understanding of the value of greenspace. Hanoi is aspiring towards the Singapore model of a ‘biophilic city' and increasingly embedding the terms of climate change mitigation and adaptation in its greenspace policies ilic city’ (e.g. Hanoi Times, 2016), whereas Fukuoka publicly emphasises the heat mitigation potential of greening via its *Green Curtain* initiative (Fukuoka City, 2016), and Taipei justifies greenspace largely in relation to flood reduction potential. There is hence at base competence in making the climate adaptation case for urban greening in each city, albeit underpinned by differing rationales.

*Opportunities for innovation, experimentation and learning* across the three cities are not a competence held by a single institution, but rather come through a mixture of top-down and bottom-up partnerships. At the level of individual projects at least, there is ample evidence of city-wide competence in this area. This includes both city-led flagship demonstration projects (e.g. the ACROS terraced garden in Fukuoka, providing biodiversity, aesthetic and cooling benefits); and also community-level projects linking communities with NGOs, academia and city governments (e.g. neighbourhood projects supported via the *Taipei Open Green* initiative; and community redevelopment projects in Hanoi led by the Arts Build Communities NGO). However, across all three cities, a lack of *ability to access long-term and self-sustaining funding* remains a challenge to up-scaling experimentation and innovation and sustaining project-based initiatives.

In terms of *participation in international knowledge-sharing* to facilitate the deployment of adaptation-focused greenspace actions, across all three cities the influence of concepts, theories and ideas from overseas on local greenspace and adaptation competence was noted. Greenspace planning in Taipei is often influenced by ideas and examples from the USA, Japan and/or Singapore due to key individuals who have overseas educational backgrounds in these countries plus connections to government in Taipei (Raco et al., 2010, Hou, 2020); academic institutions involved in greenspace planning committees in Fukuoka have prior knowledge exchange with Germany on urban climatological planning, albeit at an academic rather than practical level (Hoschele et al., 1995); and Hanoi has recently engaged in knowledge exchange with Singapore on tree management (Yarr and Nguyen, 2019) and Seoul on public engagement (ICLEI-SEAS, 2020). It was noted - especially in Taipei and Hanoi - that such concepts require localisation if they are to be effective. For example, the intensity of rainfall is much higher in monsoon areas than in temperate climates, meaning greenspaces alone cannot deal with stormwater without considering hydraulic engineering. This led to debates between planners and engineers in Taipei when low-impact development strategies were introduced into urban land use plans. In Hanoi, problems have arisen due to plans being produced by international consultants with limited knowledge of the local context.

*Linking actions with socio-economic development* takes a prominent, albeit differing, form in each city. Hanoi faces rapid expansion whereas Taipei and Fukuoka have regeneration efforts, meaning developers have significant influence in determining the future composition of all three localities. In Hanoi, the pace of urban development is such that private developers such as Gamuda and VinGroup are now key in providing publicly-accessible greenspaces around their flagship residential developments such as EcoPark. In Taipei, whilst recent city-led greenspace efforts were evaluated more positively than previous efforts such as *Taipei Beautiful* that arguably granted too much power to the private sector, developers can still have a significant bearing on the fate of community greenspaces through enacting new construction projects on vacant lands that have been temporarily used by nearby communities in the interim (Shih, 2020), or by influencing the shape, location and character of greenspaces in relation to new buildings. In Fukuoka, despite the presence of strict planning regulations to preserve existing green spaces, attempts to link greenspace in new projects to socio-economic development have been frustrated by difficulty in passing laws aimed at ensuring new developments support greenspace and adaptation.

### 5.3 Availability, synthesis and use of knowledge

For *data to support evidence-based decision-making,* respondents in each city felt there was adequate data on localised *climate change* effects (e.g. flood hazard maps, localised future climate predictions). Yet interviewees in each case believed there remained a need for basic data into *greenspace* at a city-wide level. Lacking specifically were a city-wide greenspace inventory for Hanoi; limited (albeit improving) understanding of the functions of informal greenspaces outside of designated city parks (Taipei); and a lack of clarity by some stakeholders over technical terminology such as ‘nature-based solutions’ and ‘green infrastructure’ (Hanoi). Fukuoka is one context where at regional level, ecosystem service thinking is at least mentioned in climate change planning (Fukuoka Prefecture, 2017). Given the emphasis placed by authorities in Hanoi and Taipei on international learning (see Section 5.2.) it is notable that these technical terms, which are prominent in international urban greening discourse, still have to take root

Nonetheless, reflecting the need for *decision-support and data management tools*, respondents saw competence in managing and accessing data as as big an issue as the presence of data itself. Governmental policies on managing data in Hanoi and Fukuoka mean different local government departments and national ministries hold different datasets that need to be integrated for a fuller understanding of greenspace in adaptation (Hanoi). Funding conditions associated with government-commissioned projects may complicate the reuse of data by the wider research community (Fukuoka). Although Taipei has a city- and country-wide ethos of open data (see e.g. data.taipei), it remains the case that green infrastructure reports prepared by commissioned consultants are not necessarily known or used for urban planning.

Similarly, for *capabilities in reaching and implementing decisions*, resources and/or institutional constraints were considered as big a challenge as the technical knowledge or capabilities of the individuals involved. In Hanoi, for instance, it was suggested that although there is good basic knowledge of climate adaptation and greenspace planning at an individual level, such people are constrained in their actions by the remit of the government departments they work in. In Taipei too, smaller projects to integrate adaptation into discrete greenspaces may reach innovative outcomes by involving planning or landscape consultants, yet land use change at a larger scale can only be considered within the periodical review of the urban plan. UUrban planning divisions may have limited understanding or awareness of strategic greenspace planning for climate adaptation (Taipei).

An area that did not come across strongly in the available data was competence in *integrating different knowledge systems*. For both Hanoi and Fukuoka, the greenspace-adaptation interface appears to be dominated by technocratic or natural science knowledge systems. Reflecting the greater opportunities for cross-sector participation in implementation in Taipei, initiatives such as Taipei Garden City, Smart Eco-City, and Shezi Island development attempt to integrate the knowledges of communities alongside technical experts in the decision-making process (e.g. Hou, 2020).

### 5.4 Civil society collaboration

There are historical and current examples of *cooperation with civil society* and *public participation* in each city, again reflecting overall political visions and governance structures in each country. In Hanoi, respondents referred to public participation in creating and maintaining greenspace (e.g. communal tree planting after the war, current NGO-led community regeneration activities); and there is dialogue with civil society organisations and NGOs on local climate change response planning through collaboration with ICLEI’s South East Asian Secretariat (ICLEI-SEAS, 2019). Yet small-scale protests over decisions such as the removal of trees to make way for metro lines support the observation of Yarr and Nguyen, 2019, Gillespie and Nguyen, 2019 that whilst there are participatory instances in Hanoi, the potential of such participation to meaningfully influence larger-scale decisions may be limited. Interviewees too suggested some citizens may be reluctant to speak freely during public consultations over greenspace or climate decisions. Fukuoka has citizen engagement initiatives at the greenspace and adaptation interface, such as citizen competitions for growing green walls (Fukuoka City, 2016) and the involvement of community organisations and the private sector in the *Flower City Fukuoka* initiative to propagate city-wide greening (Fukuoka City, 2020). Yet, again, there have been criticisms elsewhere in Japan that whilst mechanisms for community participation do exist (Puppim de Oliveira and Fra.Paleo, 2016), communities’ inputs are limited to superficial matters and do not inform more profound changes to plans. Civil society’s role in Fukuoka for greenspace and adaptation matters thus appears marginal, whereas in Hanoi civil society organisations have a more oppositional role.

Whilst Taipei is not immune from the issues raised above, responses suggested it came closest to *effectiveness in participation*. What is distinct about Taipei, perhaps reflecting a broader turn in the city towards e-participation (Fan, 2020) is the breadth of public participation channels. These include consultations with neighbourhood heads, public hearings, opportunities for local communities to propose policy white papers to the city government, and the creation of an e-platform to digitise information which can be established by governments or by local societies. For example, for the Taipei Garden City programme, the city government has created a Garden City Bank website and related Facebook page; and local societies also operate their own shared spaces for discussing policies and Facebook pages to engage wider users online.

### 5.5 Ethical and justice issues

A foundation for competence in *understanding access to key greenspace and adaptation assets* exists in each case in the form of basic data to assess the distribution of formal parks and greenspaces. These include inventories of greenspaces; indicators for greenspace per capita; and standards for accessible greenspace. However, in an adaptation context, there is still an emphasis on accessibility of greenspace rather than attention to distribution of greenspace functions (Taipei and Fukuoka); and a heavy reliance on greenspaces provided by the private sector such as the new EcoPark development in Hanoi, leading to claims of green gentrification and unequal access to the benefits of greenspace assets across society (Environmental Justice Atlas, 2015).

For competence in *understanding differences in vulnerability across society and space*, across all three cities much rests on the availability and accessibility of appropriate socio-economic data – and skills within local government to turn such socio-economic data into appropriate vulnerability assessment. In Hanoi, for example, vulnerable areas are calculated at ward level based on surveys conducted every five years; whereas socio-economic status (Taipei) and age (Fukuoka) are discussed as potential data sources to understand vulnerability. Yet existing disaster prevention and response programmes (such as those in Taipei and Fukuoka as outlined in Section 5.1.) do not necessarily consider land use or greenspaces. An example of efforts to translate such data into vulnerability assessments can be seen in Taipei, where there are ongoing projects to map hazardous areas for disaster prevention and prepare local governments to respond and integrate these into urban plans. An additional noteworthy factor was raised by respondents in Fukuoka, who explained that cultural sensitivities around publicly discussing issues such as poverty and marginality may act as a barrier to explicitly targeting interventions towards those most at risk.

An area of difference between cases with regard to normative competence is *explicit consideration of justice in the cities’ greenspace and adaptation plans*. These differences appear closely linked to historical social context. In Hanoi, interviewees summarised that for the last 50–60 years the ratio of greenspace has been planned under socialist ideals that everyone should be equal, but that these ideas are now coming under pressure from private sector development facilitated by ‘Doi Moi’ economic reform, which transformed Vietnam to a decentralised and privatised development model (Fan et al., 2019). The guiding principle of a ‘liveable environment’ in Fukuoka, meanwhile, can arguably be traced back to environmental justice issues in the wider Kyushu area in the 1960s and the associated desire to improve environmental quality in the public interest (Mabon et al., 2019b). In Taipei, allocating greenspaces to address environmental justice is complicated by the need to follow land use zones designated in the urban plan, which is over 40 years old and could be politically risky to change. The ongoing periodical review of urban planning in Taipei is a potential mechanism for mainstreaming associated issues to a higher-level land use plan, but the extent to which justice is considered in greenspace plans varies by case. The Eco-Shezi island proposal supported by the government in Taipei is, for instance, criticised for its neglect of local people and risk of green gentrification (Pan, 2018, Mabon, 2020); whereas practitioner, academic and citizen-led moves to appropriate vacant land for urban greening in Taipei were in part motivated by the pro-democracy Sunflower Movement of 2014 (Hou, 2020).

There are differences between cases in *measures to reduce inequalities and/or benefit the most vulnerable at the adaptation-greenspace interface*. Different organisations or partnerships are responsible for putting such measures into practice, although municipal governments play a limited role. In Hanoi, for instance, the Canadian NGO Healthbridge has been involved in work to support the engagement of one particular group - young people – with parks (Hanoi Youth and Public Space, 2015). Taipei again has collaborative approaches, such as an open platform for application for urban greening projects, with priority for communities who may benefit most from additional resourcing. What is worth noting from the Taipei case, however, is that even if measures are targeted at vulnerable communities, the capacity of communities themselves to participate in initiatives may be limited.

## 6 Discussion

We organise the Discussion around two challenges raised at the start of the paper. One is to use empirical observations to demonstrate why each competence area matters in facilitating adaptation through urban greenspace (after Wiek et al., 2011). The second is to understand what competences may look like outside of a Western context (Perez Salgado et al., 2018), and how understandings of nature-based adaptation may be ‘localised’ or ‘provincialised’ (Affolderbach et al., 2019, Chang et al., 2020) when applied across and between subtropical Asian settings. As a precursor, Table 5 summarises the key outcomes of our analysis.

**Table 5:** Summary of empirical evaluation of competences

Competence area	Why it is necessary based on empirical observation	Key tensions between cases	Illustration of competence in practice
Setting goals, targets and outcomes through policy and leadership	Gives vision for local governance actors to refer to; leadership key for setting vision and driving it to realisation.	Superficial and aesthetic greening initiatives, versus limited moves towards networked and functional greenspaces; communities of champions most apparent in Taipei.	Being able to identify opportunities to embed greenspace into other climate adaptation and urban planning actions; and connect discrete projects to a city-wide vision.
Competence in defining, developing and realising pathways towards expected outcomes	Need to go from exemplar or piecemeal projects towards broader, sustained initiatives and networks, drawing in international learning and private sector where appropriate.	Knowledge/learning comes from different international contexts across cities, and is diffused in different ways within city cases. Also differing roles for private sector in greenspace between contexts.	Identify policy and practice spaces where international networking and knowledge can gain traction from bottom-up; mobilise networks including pragmatic engagement with private sector.
Availability, synthesis and utilisation of knowledge	Understand how to use knowledge *institutionally* (i.e. beyond individual expertise) to facilitate adaptation via greenspace functions.	International concepts understood and interpreted differently in different cities, e.g. ‘green infrastructure’ used in Hanoi to mean low-carbon infrastructure; yet starting to be interpreted in Taipei and Fukuoka as an ecological network.	Develop common understandings within city context of what approaches such as nature-based solutions and green infrastructure mean, and how they can be deployed appropriate to local context.
Civil society collaboration	Different governmental sectors and policies can have contradictory impulses, non-government actors (civil society) may influence what outcomes are attainable.	Taipei – new and flourishing democracy with emphasis on participation; Hanoi – authoritarian with oppositional role for civil society; Fukuoka – top-down committee-driven with peripheral role for civil society.	Steering stakeholder diversity within the confines of what is possible in different political systems.
Ethical and normative	Failure to address normative issues can lead to contestation, disruption, delay; also moral imperatives to avoid harm.	Cultural and political backdrop shapes norms. Hanoi socialist, emphasising equity (but eroding?); Taipei new democracy with drive of key actors to ‘better’ society via greenspace; Fukuoka, where vulnerability seen as source of shame, limits explicit normative discussion?	Identify places and opportunities to integrate equity into existing planning processes appropriate to local context; adapt process and recognition justice to different systems.

### 6.1 Setting goals, targets and outcomes through policy and leadership

Each of the three cities has some kind of greenspace plan and to a lesser extent climate adaptation plan, and indeed respondents in each city (including those outside of municipal government) referred to these plans and policies as a guiding principle for the greenspace and adaptation actions they engaged with. This supports the assertion in the sustainability competences literature that spatial planning or efficient use of space (Holtz et al., 2018, Kerry et al., 2012) is an important part of competence in laying out a vision (Wiek et al., 2011, MacDonald et al., 2020); and reflects empirical findings from other geographical regions on the value of plans and visions in coordinating different actors to work towards adaptation via urban greenspace (Gradinaru and Hersperger, 2019; Hansen et al., 2019, Hislop et al., 2019).

Nonetheless, findings from Hanoi and Fukuoka in particular indicate that competence in setting a spatial vision and promoting city-wide urban greening initiatives is not in itself sufficient to support adaptation. Municipal rhetoric on ‘green’ or ‘garden’ cities – Hanoi’s *One Million Trees* programme; and Fukuoka’s *Flower City Fukuoka* initiative – was criticised for being superficially focused on abundance and urban beautification, as opposed to climate adaptation or the resilience of citizens to environmental stresses. In both Hanoi and Fukuoka, respondents were also sceptical as to whether greenspace visions could be realised in the face of real estate development pressures. Taipei however offers insight into how high-level visions may translate into meaningful benefit to citizens. Taipei’s Garden City programme, for example, has engaged with climate adaptation and was broadly evaluated positively in terms of affecting tangible change. What makes the Taipei Garden City programme comparatively effective is that champions supporting the initiative spanning municipal government, academia and NGOs were able to influence policy development at city level and then support its implementation by linking different sectors and organisations. The Taipei Garden City initiative thus illustrates that a policy and planning vision needs to be driven by champions with competence in identifying key leverage points in a system (Wiek et al., 2011); linking different knowledge systems (Jacobsson and Karltorp, 2012); and turning policy rhetoric into tangible interventions (Perez Salgado et al., 2018).

Our findings therefore partially make the case for setting goals, targets and outcomes through policy and leadership as a necessary competence for adaptation via urban greenspace. On one hand, across all three cities, policies and plans do offer a high-level coordinating vision to guide urban greening actions. Yet for these plans and policies to translate into tangible actions, they may need to be driven forward by champions with a breadth of competences in navigating the policy and governance landscape. However, one may also question the extent to which the competences of key individuals within the policy process (as seen in Taipei) are a substitute for broader powers and capabilities at the institutional level to turn policy rhetoric into reality.

### 6.2 Defining, developing and realising pathways towards expected outcomes

As an illustration of ‘getting things done’ (Wiek et al., 2011), all three cities have examples of small-scale and/or community-level innovation and experimentation at the interface of greenspace, resilience and adaptation. These initiatives reflect the niche experiments and social learning which are valued in existing urban sustainability competences (Holtz et al., 2018) and nature-based adaptation (Frantzeskaki et al., 2017; Castán Broto and Bulkeley, 2013) scholarship. Yet competence in initiating these niche experiments comes from different sources in each case: civil society organisations and international organisations in Hanoi; local government in Fukuoka; and a combination of local government, civil society and community in Taipei. Of the three, Taipei perhaps comes closest to upscaling beyond discrete individual projects (as advocated by Bai et al., 2018) through the presence of coordinated city-wide networks such as *Taipei Open Green*. There is also difference in how experience and knowledge of urban greening practices from other countries is localised in each city. Understanding what makes the adoption of practices from elsewhere effective matters given increased scholarly attention to policy mobilities for green cities (Affolderbach et al., 2019) and high-level advocacy of city-to-city networking for resilience (Bai et al., 2020); and also the emphasis given to contextualising knowledge within the competences literature (Solís-Espallargas & Morón-Monge, 2020). In Hanoi, urban greening practices for resilience appear largely imported via structured top-down initiatives, such as the ICLEI *Ambitious City Promises* link-up driven by Seoul Metropolitan Government, and international consultants offering urban planning advice, leading to piecemeal adoption. In Taipei, on the other hand, experiences and insights are imported and localised from the bottom up, focusing on Seattle and Seoul due to the interpersonal networks of individual practitioner-academics. Competence in international networking hence may need to originate in actors *within* the locality rather than coming from outside if it is to support effective greenspace adaptation interventions.

Competence in ‘getting things done’ and making interventions happen (Perez Salgado et al., 2018; MacDonald et al., 2020) for adaptation via urban greenspace in a subtropical Asian city setting may hence require the presence of institutions capable of linking discrete projects together to up-scale innovations. Taipei’s comparative success points towards the value of locally-based individuals or institutions who have an in-depth knowledge of ‘what works’ elsewhere (and why), yet also understand the local policy and practice landscape in a way that allows them to identify leverage points to shape local policy and planning processes. Moreover, reflecting the need to collectively design and implement transitions (Wiek et al., 2011), our cases also indicate that competence in realising pathways may need to include competence in working with the private sector to realise adaptation via urban greening. The rapid expansion of Hanoi means that the private sector has a significant role in the provision of new greenspace within new real estate developments; and in Taipei, urban regeneration means developers can still influence the preservation or loss of existing greenspace through their decisions to enact new projects (e.g. Shih, 2020). The challenge across the different cases is thus to develop competence for adaptation via urban greening in a way that recognises the pragmatic importance of collaborating with the private sector to ‘get things done’, yet does not alienate other institutions involved in initiating and upscaling experiments and innovations.

Our findings hence show two reasons why competence in defining, developing and realising pathways towards expected outcomes is important. One is that competence in localising international experience, and connecting small-scale community-level experiments, is strongly present in Taipei as the case study city making the most progress towards networking and learning from practical actions. The second is that as private sector developers hold significant sway over greenspace provision or preservation in at least two of the cases, there is a real need to include a breadth of actors – not only municipal governments and communities - in the collective design of pathways towards greenspaces for adaptation.

### 6.3 Availability, synthesis and use of knowledge

In each city, respondents believed there were local researchers, and individuals within municipal governments, with good competences in understanding risks from climate change. Our interviewees also felt the underpinning climate data in each case was sufficient to guide responses. This is notable given the continued emphasis on the need for technical capacity building in climate adaptation for subtropical Asian cities (e.g. Friend et al., 2014). However, competence in systems thinking requires not only understanding but also *responding to* harmful effects (Wiek et al., 2011). Respondents believed there was much less indication of data and knowledge being used to support evidence-driven responses. The issue is thus perhaps one of being able to apply existing knowledge competences to influence policy and practice, as well as acquiring new knowledge. This finding shows the limitations of knowledge competences when they are held at the individual or small group level (Jacobsson and Karltorp, 2012, MacDonald et al., 2020), especially for issues such as climate adaptation through greenspace which require different governmental sectors to work in collaboration, and for actors to be able to synthesise different data sources held by different bodies.

Indeed, in cases where knowledge-driven policy and practice interventions were evaluated positively, such as the Smart Eco City and Garden City initiatives in Taipei and the formation of an integrated mitigation and adaptation plan in Fukuoka, respondents suggested it was because key individuals’ knowledge competences were supported with competences in understanding how to enact interventions in practice (Perez Salgado et al., 2018). The value of individuals and departments who can combine techno-scientific knowledge with socio-political nous is of course not limited to subtropical Asian city contexts (Roberts et al., 2020). However, given the prevalence of top-down and/or siloed governance modes in subtropical Asian contexts (Dobbs et al., 2014), the need for competence in making knowledge and data work across divisions may be equally important.

Additionally, respondents in each city felt there was a lack of comprehensive knowledge and data to understand greenspace functions, and the role of these functions in climate adaptation, at a city-wide level. It is important not to be overly critical of this lack of knowledge, given that thinking in terms of a city wide urban ecosystem delivering resilience-building functions is a very new approach in planning globally (Douglas et al., 2021). Nevertheless, in a tropical zone context, the species and configurations of greenspaces may be very different to temperate climates (Song et al., 2017, Giridharan and Emmanuel, 2018). There may thus be limits to the usefulness of concepts imported from elsewhere if applied without local assimilation. Given the prominence granted to international learning and knowledge-sharing in Hanoi and Taipei especially, knowledge competence for adaptation via urban greening in a subtropical city thus requires ability to understand what is going on in the world (Kerry et al., 2012) and seek out information (MacDonald et al., 2020) but also to ‘provincialise’ new international concepts (Chang et al., 2020) to reflect how urban nature functions in the local context.

We thus partially make the case for competence in accessing, synthesising and utilising knowledge. On one hand, it is true that climate change poses real risks with the potential to cause harm, and that there is a need to understand how tropical ecosystems may function differently to those in temperate climates that take prominence in much international rhetoric to date. But our findings also indicate that knowledge competences are only likely to be effective if strongly linked with competences in enacting interventions (Perez Salgado et al., 2018; MacDonald et al., 2020) and ‘getting things done’ (Wiek et al., 2011). These challenges may be especially pronounced in subtropical cities where local governments remain strongly segregated – like Fukuoka – or lack fora where data and knowledge may be synthesised, as is the case in Hanoi.

### 6.4 Civil society collaboration

Civil society collaboration is an area in which the difference between local contexts comes across strongly. Given that Wiek et al (2011) see this kind of interpersonal competence as a ‘cross cutting’ skill set influencing other competence areas, these differences are worth discussing. The wider turn in Taiwan towards e-participation and e-democracy (Fan, 2020) is reflected in the increasing instances of direct engagement by municipal governments in Taipei with communities, academics and NGOs on urban planning. These do not focus on adaptation per se, but may facilitate community resilience more widely, yet have in cases (e.g. Shezi Island redevelopment) been criticised as a superficial mode of participation. In Hanoi, meanwhile, the role of civil society actors is one of either opposition (Gillespie and Nguyen, 2019), or of engaging in municipal climate change governance initiatives through intermediary institutions such as ICLEI. Despite Hanoi’s authoritarian context, it is also not necessarily the case that civil society actors are completely disempowered. There is empirical evidence from elsewhere (e.g. Coe, 2015) to indicate civil society action in Hanoi can influence public debate and shape municipal greenspace decisions.

When compared to competences scholarship from ‘Western’ settings, Taipei as a relatively new and vibrant democracy probably comes closest to demonstrating competences in network building (Holtz et al., 2018) and understanding, comparing and critically evaluating different positions (Wiek et al., 2011). However, given the breadth of political contexts in the sub-tropics from democracy to authoritarian (Dobbs et al., 2014, Han, 2017), it may be unfair to compare collaborative competences directly across political contexts. This is not to say that collaborative competences are ‘easier’ in a democratic setting, simply that competences in collaboration may look different across different social and political contexts. In Hanoi, for instance, it might be that NGOs and civil society are important in steering stakeholder diversity and facilitating action towards practical decision-making on greenspaces, but that this happens outside of formal government channels (Coe, 2015, Gillespie and Nguyen, 2019). Future research into competences for innovation and experimentation in subtropical Asian contexts may also wish to consider lessons that can be learned from greenspace and adaptation in post-Socialist states in Europe, which can give insights into greenspace and adaptation development in relatively new democracy contexts with differing institutional and governance histories (e.g. Badiu et al., 2019).

In Taipei, which has a vibrant civil society, and Hanoi, which has a small but growing civil society sector, competence in collaboration offers an alternative pathway to protracted opposition and confrontation. If the aim is to facilitate practical adaptation actions via greenspace, these examples thus ‘make the case’ for competence in civil society collaboration. However, it is more difficult to understand what collaborative competence may look like in practice across different political contexts. Relations of trust between municipal and civil society actors, and local norms about how decisions ought to be made, influence the nature of collaboration competences. The growing civil society movement in Hanoi compared to the very marginal presence of NGOs in Fukuoka also shows that opportunities for collaboration may not necessarily be greater in more established democratic contexts. In a subtropical Asian city setting, it may thus be best to understand collaborative competence within a municipality as steering stakeholder diversity (Perez Salgado et al., 2018) *within the confines of what is possible in different political systems*.

### 6.5 Ethical and normative

The underlying ethical and normative issues faced in the three cities – potentially unequal distribution of greenspace across districts and wards, dominance of powerful private sector interests in policy processes, and questions around who is recognised in greenspace and adaptation debates – are not radically different to those seen in North American and European research (e.g. Haase et al., 2017, Keeler et al., 2019). However, between the city contexts, the social, political and cultural backdrop leads to notable differences in what municipalities’ ‘acquired normative knowledge’ (Wiek et al., 2011) looks like and how ethical principles are explicitly applied in practice (Solís-Espallargas & Morón-Monge, 2020). Indeed, Moser (2020) believes it is especially important to understand normative issues across the diverse political contexts in which much new urban development is happening.

Again, reflecting Dobbs et al’s (2014) finding that democracy has mixed effects on the benefits people derive from urban ecosystems, it is not necessarily the case that more ‘liberal’ or ‘democratic’ governance systems facilitate greater consideration of ethical and normative issues. In Hanoi, for instance, respondents suggested Vietnam’s socialist system was traditionally quite well-disposed to top-down equitable distribution of greenspace, but that this had been weakened post-Doi Moi with bigger focus on economic development and associated urban development pressures (Fan et al., 2019). Recent claims to procedural injustice around tree-felling in Hanoi (Gillespie & Nguyen, 2019) indicate these normative competences may have been eroded. In Taipei, some of the small-scale experimentation that has emerged with strong municipal support for urban farming and community resilience-building has its roots in the pro-democracy ‘Sunflower Movement’ and the appropriation of vacant urban spaces (Hou, 2020). Yet, in Fukuoka, despite the early engagement of an epistemic community with interest in the liveability of the urban environment (Mabon et al., 2019b), societal norms around shame and poverty were argued by respondents to make explicit discussion of vulnerability and climate justice challenging. Comparing Hanoi to Taipei may, however, show that greater democracy leads to stronger competence in dealing with dissent and unbalanced power relations, in a way that leads to more productive and consensual outcomes (Wiek et al., 2011).

Our findings make the case for ethical and normative competences in that the examples of greenspace deployment providing the most benefit to communities – such as rapid proliferation in Hanoi in the post-war period and experimentation in Taipei following the Sunflower Movement – closely link to the explicit application of ethical and normative standpoints (Solís-Espallargas & Morón-Monge, 2020). However, what Hanoi and Taipei especially show us is that normative competences are not static over time, and that ideas of who ought to benefit from greenspace and how this ought to be achieved may be significantly influenced by the overarching political context. Indeed, reflecting Kusno (2011) on how middle classes were able to capture the emergent urban greening movement in Jakarta, further research in subtropical Asian city contexts may wish to assess the extent to which normative competences can continue to bring benefits to citizens from greenspaces in the face of shifting political priorities.

## 7 Conclusion

In this paper, we evaluated climate change adaptation via greenspace in three subtropical Asian cities with different governance and development contexts – Hanoi, Taipei, and Fukuoka. To do so, we used the lens of competences, which we interpreted as city governments and the individuals working within them (as well as the wider governance system) having both the skill sets and the policies and legislation to reach greenspace adaptation decisions appropriate to the trajectory of the locality. By evaluating policies and scholarly literature and interviewing practitioners and experts in each city, we sought to build on the growing body of literature around the social and political dimensions of climate change adaptation via greenspace, especially responding to calls for greater empirical research in this area in tropical zone and/or non–‘Western’ contexts. Conceptually, we also aimed to further scholarship on the practical value of competences in environmental decision-making, and to contribute to ideas of how international rhetoric on nature-based adaptation becomes localised to subtropical contexts. In this regard, we conclude with three critical challenges identified across the case study cities, where strong competences are particularly important. One is the importance of individuals and/or institutions able to work across boundaries and get buy-in for adaptation actions in the presence of inflexible municipal policy and funding structures. A second is the rapid nature of development and expansion (or at least renewal) in subtropical Asian cities, which may place additional pressure to balance greenspace and adaptation with socio-economic development pressures and risk the kind of ‘green climate gentrification’ attracting concern in Europe and North America. Third and final, as per Escobedo et al. (2019), ‘green adaptation’ and associated terminology have strongly Western origins. Our findings indicate that even in the absence of terminology such as ‘green infrastructure’, ‘nature-based solutions’ and ‘ecosystem services’ currently favoured by international agenda-setting organs, all three cities have to an extent engaged with adapting to climate change via greenspace. As the nature-based adaptation agenda advances globally, a key challenge will hence be to understand how international best practices become ‘localised’ and are integrated with existing local knowledge of greenspace and climate.

## Funding

The data on which this paper is based was collected through: (a) Wellcome Trust Seed Award in Humanities and Social Sciences (205764-Z-16-Z) (LM, WYS); (b) Royal Society of Edinburgh-Ministry of Science and Technology Joint Research Project (MOST 106 -2911-I-130 -502) (WYS, LM); (c) Scottish Funding Council Global Challenges Research Fund funding allocated to Robert Gordon University and assigned to LM; and (d) Scottish Funding Council COVID-19 Research Uplift funding allocated to the Scottish Association for Marine Science at the University of the Highlands and Islands and assigned to LM. No funder had any influence over the design, execution or dissemination of the research.

## Declaration of Competing Interest

The authors declare that they have no known competing financial interests or personal relationships that could have appeared to influence the work reported in this paper.
