# Supplementary material for: Urban greenspace as a climate change adaptation strategy for subtropical Asian cities: A comparative study across cities in three countries
Source: Glob Environ Change. 2021 May;68:102248. doi: 10.1016/j.gloenvcha.2021.102248 (PMC8164163; doi:10.1016/j.gloenvcha.2021.102248)
Supplement: Supplementary Data 1 [file mmc1.pdf]

MABON, L, AND SHIH, W-Y (2021) 'URBAN GREENSPACE AS A CLIMATE CHANGE ADAPTATION STRATEGY FOR SUBTROPICAL ASIAN CITIES: A COMPARATIVE STUDY ACROSS CITIES IN THREE COUNTRIES' GLOBAL ENVIRONMENTAL CHANGE DOI 10.1016/J.GLOENVCHA.2021.102248

SUPPLEMENTARY DATA: INVENTORY OF UNDERPINNING EVIDENCE FROM POLICY/DOCUMENT REVIEW, INTERVIEWS AND EXISTING RESEARCH

		**HANOI**	**TAIPEI**	**FUKUOKA**
1. Goals, targets and outcomes through policy and leadership	What are the key policies and legislation mentioning greenspace and adaptation?	Hanoi Urban Planning Masterplan to 2030 and Vision to 2050;<br><br>Law of Planning - backbone of general planning for whole nation;<br><br>Six levels of city in Vietnamese planning law – larger cities should have higher greenspace proportion (in theory). Ministry of Construction sets criteria for urban greenspace (interview with urban planning academic; interview with Vietnamese planning expert; Pham et al, 2013). Design standards of "Greenery planning for public utilities in urban areas (TCVN 9257: 2012)", trees in public greenspaces - which include public greenery, parks, green lands and gardens, and green trees - should be allocated proportionally in relation to the population size of a city.<br><br>No whole-city strategy for parks or public spaces – but there are	National Spatial Plan (2018)<br><br>Adaptation Strategy to Climate Change in Taiwan (2012)<br><br>National Climate Change Adaptation Action Plan: Land Use Sector (2013-2017/2018-2022)<br><br>2012 Climate Change Adaptation guidance for Taiwan<br><br>Water Act (2019)<br><br>Taipei City Climate Adaptation Plan (2012)<br><br>Taipei Urban Plan: periodical overall review of Urban Planning (2018/2019) – includes disasters and hazard mapping (especially for flooding); and minimum greenspace size-population standards.	National Plan for Adaptation to the Impacts of Climate Change (2015)<br><br>National Climate Change Adaptation Act (2018) – mandates local authorities to prepare climate change adaptation plan.<br><br>Fukuoka City New Green Basic Plan (2009) – includes potential of greenspace for rainfall and heat mitigation measures.<br><br>Fukuoka City Climate Change Countermeasures Action Plan (2016) – core climate adaptation and mitigation policy for Fukuoka City. Mentions green walls as heat mitigation strategy.<br><br>Fukuoka City Urban Planning Masterplan (2014) – core land use plan for Fukuoka City.

		district strategies (interview with urban planning academic); no consideration of climate change in Masterplan (interview with climate change researcher)<br><br>One Million Trees Programme – plant one million trees towards attaining goal of providing up to 10-11 m2 of green area per person by 2020;<br><br>People's Committee of Hanoi Decision No. 1745 (2012) – includes awareness raising regarding climate change, particularly its impacts on people's daily lives, increasing the investments on green and smart infrastructure, and improving the people's capacity to adapt to the changing climate. 79 tasks to be completed in 11 areas of the city with 12 main programs and 82 specific projects (ICLEI-SEAS, 2020)	Integrated Flood Control/Management plan and policies since 2003 - encourage use of greenspaces to manage runoff.<br><br>Since 2005 major cities in Taiwan has prepared "Regional Disaster Prevention and Response Plan", which prepare Taipei with various kind of hazard maps. The work has further decentralised to enable building resilience in local communities.<br><br>Smart Ecological Communities project (since 2015) - integrates green infrastructure (more recently garden city) and disaster reduction.<br><br>Taipei Sponge City initiative (Taipei City Hydraulic Engineering Office, Public Works Department, n.d.)	Influences greenspace, but limited explicit engagement with climate adaptation.<br><br>Fukuoka City Central Park Basic Plan (2019) – greenspace plan for central Fukuoka.<br><br>Flower City Fukuoka Programme (2018-present) – cooperation with local business and NGOs to facilitate small-scale greening across city.
	How effective are these policies perceived to be?	Policy effectiveness of masterplan very weak (interview with economic development research institute);<br><br>None of the former plans for Hanoi (1998, 2003, 2008) reached full	Taipei 2050 as vision, including green infrastructure vision – but this has no budget and not realised. Also need for landscape law to reduce conflict and integrate with open space	More radical elements of urban climatological planning unlikely to be implemented (e.g. creation of wind corridors), as would necessitate knocking down buildings (interview with

		implementation (Leducq & Scarwell, 2018)<br><br>Greenspace and adaptation issues mentioned in documents, but no tools to implement or regulate (interview with greenspace researcher; interview with planning consultant);<br><br>Actions to mainstream climate change into planning in Hanoi, but this is largely 'on paper' and not in practice (interview with Vietnamese planning expert);<br><br>Smaller policy and practice gains, for example from 2015 Hanoi People's Committee ordered construction projects to limit number of trees being cut down, move towards relocating trees rather than cutting down (group discussion with researchers)	system, but little legislative support (landscape architect interview)<br><br>Understanding that policies and laws can be way to promote green infrastructure, but need regulation, integrate information into laws, and change policies (landscape architect interview)<br><br>Political cycles frustrating – if government changes, need to renegotiate with new government (landscape architect interview)<br><br>Different mandates for functions across different government sectors, e.g. Parks and Streetlights water focus, CO_2 mitigation mandate of Environmental Protection Bureau (Taipei City Parks and Streetlights interview)<br><br>Already lots of projects in Taipei Government with comprehensive vision, problem is no policy support tool for implementation (Taipei City Land Administration interview)	environmental research institute);<br><br>Similarly, inclusion of greenspace within building codes and developments difficult to implement, as not supported by developers/wider city government (interview with Fukuoka City Green City Promotion Division).

			Work mainly on case-by-case basis for greenspace in urban development, no overall plan (Taipei City Urban Development interview; academic urban planning committee member interview)	
	What mechanisms exist for integrating across sectors, and how effective are these perceived to be?	Over 20 governmental departments (corresponds to number of government ministries) – very hard to get people to sit down together, so cooperation is weak (interview with climate change researcher);<br><br>Each sector has plans, but separated and no connection between sectors – over 19,000 plans in Vietnam, so some overlap inevitable (interview with Vietnamese planning expert).	2012 national climate adaptation guidance helps to work across silos, by providing more comprehensive coverage of areas to be taken into account and facilitating more cross-sector discussion (greenspace planning consultant interview)<br><br>Different government offices have different priority – Parks and Streetlights Office (primarily responsible for the implementation of parks and greenspaces designated in urban plan) lower priority and needs higher role to cover more issues e.g. conservation areas, river fronts (which are currently managed by different office) (landscape architect interview)<br><br>Challenging to integrate urban plans and greenspace/green	Limited collaboration between government departments – although attempting to improve via annual forums (interview with Green City Promotion Department);<br><br>However this problem common across Japanese cities for adaptation and not limited to Fukuoka (Baba et al., 2017; Hijioka et al., 2016)

			infrastructure strategies – areas where there are linkages are based on personal contacts of individuals? (landscape architect interview)<br><br>Valuation for urban development system values construction more, so can affect green infrastructure which is already there (Taipei City Land Administration interview)<br><br>Planning committee of periodical review can raise CCA issues, but developers do not necessarily give a tangible response (academic urban planning committee member interview).	
	What mechanisms exist for integrating different levels of government, and how effective are these perceived to be?	Local government has weak capability – not because of knowledge or technology, but because of cooperation and coordination. Ministries at provincial level report to national level (interview with climate change researcher);<br><br>National assembly meets only twice a year, and need to prepare documentation etc beforehand – so can take time to get approvals for	Parks and Streetlights office traditionally second-level office, so what they can do for designated parks and greenspaces is decided by urban plan – which did not necessarily accurately portray nature of greenspaces. Now feeding back into urban plan review, attending meetings and reviewing location of greenspaces to feed back to	Different local government departments report to different national ministries – silo effects (interview with Fukuoka City Green City Promotion Department)

		municipal-level actions in Hanoi (interview with international aid organisation)	consultants (Taipei City Parks and Streetlights interview)	
	Who is leading the agenda on greenspace for climate adaptation locally? Are there any 'champions' within the city?	Vision from Hanoi People's Committee for One Million Trees programme<br><br>Recent policies reflect strong political will to enhance Hanoi's greenery due to support from the mayor (Leducq and Scarwell 2018)	Mayor Ko Wen-Je – whilst not important for adaptation/greenspace per se, has vision for citizen participation within Taipei which includes greenspace decisions (Parks and Street Lights Office interview). Mayor also open-minded, so if justification for institutional change is made well it can be discussed at higher level (landscape architect interview)<br><br>Po-Hung Liu/Classic Design and Planning – facilitation of forums for connecting landscape experts, planners, communities etc<br><br>Vice-Mayor important for driving forward agenda on adaptation and planning (Vice-Mayor majored in urban planning) (urban planning consultant interview)<br><br>Previous mayor came from Environmental Protection Association – good	Community of academics in local institutions (e.g. Kyushu University, Fukuoka University, Kyushu Environmental Evaluation Association) working at science-policy interface e.g. planning committees on greenspace for climate adaptation (Mabon, Kondo, Kanekiyo, Hayabuchi, & Yamaguchi, 2019);<br><br>Role of city mayor in raising profile of Fukuoka City as smart / innovation city, including internet of things for disaster prevention (but see below – focus on economic development conflicts with environment/planning imperatives?)

			understanding of environmental issues (urban greenspace planning academic interview)<br><br>*But* perhaps rely too much on champions and good timing, when what is needed is science-based information and collaboration with academia to make stronger case (landscape architect interview)	
2. Defining, developing and realising pathways from the present towards envisioned outcomes	What are the main rationales/justifications that are given the city for greenspace provision?	Tree planting as culturally-significant activity, established by Ho Chi Minh (interview with urban planning academic);<br><br>Vision of developing Hanoi as 'biophilic city' like Singapore (interview with urban planning academic)<br><br>Inclusion of green corridors and greenspace proliferation in Hanoi Capital Construction Master Plan to 2030 and Vision to 2050	Key aims of Taipei greening: liveability/walkability; ecologicalisation; children's playgrounds; 'happy garden city'; colourful flowers through seasons; bright light dazzling city (Taipei City Parks and Streetlights interview)<br><br>Green infrastructure – difficult words for citizens to understand, GI vs 'parks and recreation' understanding? (urban greenspace planning academic interview)<br><br>Key adaptation focus of formal greenspaces: stormwater management and 'sponge city', emphasis on reducing runoff and discharge from parks (Taipei	Provision of 'comfortable living environment' for citizens has been core motivation over several decades (e.g. Fukuoka City Greenspace Plan, 1999; 2009);<br><br>However more recent awareness of climate adaptation benefits (especially cooling) of greenspace, and utilisation of adaptation rhetoric in justification for greenspace preservation/proliferation (e.g. Fukuoka City Climate Plan, 2016)

			City Parks and Streetlights interview)	
	What opportunities are there for innovation, experimentation and learning? Are there examples of flagship/exemplar projects, or even smaller-scale experimentation?	One Million Trees Programme – plant one million trees towards attaining goal of providing up to 10-11 m2 of green area per person by 2020;<br><br>AREP Ville demonstration park in Hoan Kiem, Greenspace AVANT public-private partnership for Hanoi greenspace – but take time to progress as important to get it right (interview with planning consultant);<br><br>Grass-roots initiatives e.g. Arts Build Communities to transform community spaces (albeit for social, not climate purposes) (interview with urban planning academic)	Taipei Open Green – small-scale activities, involvement with local people (landscape architect interview)<br><br>Government often ask for 'exemplar' or showcase park which they can show to others (landscape architect interview)<br><br>Da'an Green Vision – 5 heads of neighbourhood engaged with green roofs, still operating (landscape architect interview)<br><br>Smart eco-community pilots – 20 pilots for developing indicators, led by Department of Land Administration and involving different government sectors, universities, and practitioners (Taipei City Land Administration interview)<br><br>Taipei Garden City Initiative.	One Person One Flower / Flower City Fukuoka – collaboration with local businesses and NGOs to promote greening actions across city;<br><br>ACROS Fukuoka – flagship terraced garden offering biodiversity conservation and limited local heat mitigation (albeit greening initially for aesthetic and not strategic reasons) (Hagishima, 2018; Jim, 2017);<br><br>Island City Central Park/Grin Grin Active Learning Space – 1000m^2 green roof area.
	Does the city participate in knowledge-sharing, both within the city and internationally?	Problem: plans can be developed by international consultants with limited local experience and knowledge (interview with climate change researcher);	Theories, concepts and practices come to Taipei informally from overseas via governments (e.g. urban integrated flood control), NPOs (e.g. farming urbanism network), NGOs and academics	Fukuoka Asian Urban Research Center – objective of raising awareness of Fukuoka internationally through provision of data relating to city

		International cooperation and knowledge-sharing – different types e.g. suggestions for benefit of Hanoi People's Committee from City of Paris; technical guidance on infrastructure projects (interview with planning consultant; interview with international aid organisation)<br><br>Collaboration with Singapore National Parks Board on tree planting and maintenance; collaboration with Seoul Metropolitan Government on citizen participation via ICLEI Ambitious City Promises (ICLEI-SEAS, 2020)	(e.g. learning from Seattle case), cases selected as they are quite successful (Taipei City Parks and Streetlights interview; grey literature); but challenge is localising to Taipei's land use and weather (landscape architect interview, Taipei City Parks and Street Light office interview)	(city government-supported not-for-profit organisation)
	Are greenspace and adaptation linked with economic development? If so, how?	Private sector getting involved in greenspace provision in Hanoi – can provide reputational benefit and be linked to housing provision (interview with urban planning academic; interview with planning consultant);<br><br>Hanoi has 'green growth' action plan, as does almost every province – but not connected to climate change actions (interview with economic development research institute)	Need to provide more on the value that can be obtained from greening, make the case for investment from government (greenspace planning consultant interview)<br><br>Issues such as development and regeneration tend to be prioritised over green infrastructure – need to embed support for GI and environment for more buy-in (Taipei City Land Administration interview)	Tenjin Big Bang (renewal of 30 buildings in central Fukuoka to promote socio-economic development) provides incentives for pro-environmental actions;<br><br>Yet greenspace/adaptation still seen as in opposition to each other in some areas (interview with environmental research institute; and with Fukuoka City Green City Promotion Division).

			Targets may be there for greenspace and climate change, but not always motivation to implement them – especially if relying on private sector-led regeneration projects which focus on profit (Taipei City Urban Development interview)	
	Is there long-term and self-sustaining funding for initiatives?	High cost to implement masterplan, not sure where money will come from (interview with climate change researcher);<br><br>Increasing role of private sector in greenspace provision e.g. Ecopark, Gamuda (interview with urban planning academic);<br><br>Difficult to know if investments are climate change-related or not (especially for adaptation) (interview with economic development research institute)	Piecemeal/project-based? Funding can come on annual basis, e.g. can give suggestions to government for future issues to address, the commission companies to do projects (greenspace planning consultant interview)<br><br>Value of people skilled in finding other sources for implementation, e.g. working with Urban Development/Parks and Streetlights who have mandate and need to deliver (landscape architect interview)<br><br>Local government tendering system a barrier to evidence-driven and effective greenspace work – go for low prices so comprehensive studies or public engagement may not be funded (landscape architect interview)	Municipal greenspace and adaptation measures to date more project-based, so challenge in sustaining outside of project periods? (interview with environmental research institute)

			Integration into urban plans, and regulations (e.g. greenspace in Water Act) increases influence and long-term sustainability of initiatives.	
3. Availability, synthesis and utilisation of knowledge	Is there comprehensive environmental data to support evidence-based decision-making? What is there, and what is missing?	Don't have complete picture of greenspace in Hanoi until now, which makes it hard to make evidence-based decisions (interview with greenspace researcher);<br><br>Need for more empirical surveys and data collection to understand where and what exactly greenspace in Hanoi is (interview with Vietnamese planning expert);<br><br>Different data about greenspaces rests with different organisations – e.g. water of lakes is Department of Agriculture and Rural Development; land is another agency; trees another – hence statistical data has lots of differences (interview with Vietnamese planning expert);<br><br>Different ministries have different mandates for collecting data – so lots of relevant data does exist, but not always easy to synthesise or know where it is (interview with economic development research institute)	Taipei green infrastructure plan – use landscape indicators/NDVI to determine which districts need more action or greening – GIS-based (greenspace planning consultant interview)<br><br>Plans related to integrated greenspace systems/eco-city produced since 1990's – important references for practitioners, but not necessarily enforced.<br><br>Key problem: already have professional knowledge, but big gap to implementation, as need to negotiate with different sectors (greenspace planning consultant interview)<br><br>Need more basic studies to underpin projects, need numbers and to define locations – can learn about theories and concepts from overseas, but need to localise information to Taipei's land use, weather	Localised climate projections from Fukuoka District Meteorological Office (Fukuoka District Meteorological Observatory, 2017);<br><br>Strategies for preserving/creating wind corridors, creating cool spots via greenspace, and identifying areas of higher temperature included within Fukuoka City New Green Basic Plan (2009);<br><br>Studies into relation between greenspace and urban thermal environment commissioned by Fukuoka City Government (2003);<br><br>*But* data from government projects not readily available for re-use or sharing under conditions of funding? (interview with research institute)

			patterns etc (landscape architect interview)<br><br>*But* perhaps rely too much on champions and good timing, when what is needed is science-based information and collaboration with academia to make stronger case (landscape architect interview)	
	What kind of decision-support tools to help non-technical officials understand greenspace and adaptation? If not, what is missing?	Committees do not have enough information on differences between greenspace, how much and where – need this to build awareness and raise priority (interview with greenspace researcher);<br><br>Knowledge not always used in practice, can come to wrong person at wrong time, hence importance of face-to-face interaction to raise awareness (interview with climate change researcher)	Prior to Taipei's climate adaptation plan, 2010 green infrastructure plan provided high-level framework for arguments to communicate with different sectors (greenspace planning consultant interview)<br><br>Periodical review of urban plans takes local research or consultant advice on, for example, green infrastructure, urban heat island effect, climate maps.	Production of climate function map focusing on effects of green space on urban heat islands with urban climate simulation system under direction of Ministry of Land, Infrastructure and Transport (Ichinose, Mikami, Niitsu, & Okada, 2003; Yoda & Katayama, 1998); also building/neighbourhood-scale studies included in New Green Basic Plan (2009).
	What are the technical capabilities (for greenspace and adaptation) of the policy-makers and stakeholders who are involved in reaching and implementing decisions?	Resources key issue for Hanoi rather than capability – there are researchers and officials in departments with good capabilities, but can lack numbers of people (interview with greenspace researcher; interview with climate	Urban Development Department and Parks and Streetlights Office are two departments associated with greenspace issues – but don't understand what climate adaptation and green	Recruitment of specialised staff within city government departments (interview with Fukuoka City Green City Promotion Division); support from expert committees (interview with academic involved in planning

		change researcher; interview with Vietnamese planning expert);<br><br>Basic problem is not competences of competence of planners and policymakers, but rather implementation process and getting developers to follow procedures (group discussion at human geography research institute involved in urban planning policy recommendation)	infrastructure are (landscape architect interview)<br><br>Need more interdisciplinary people to link different aspects such as civil engineering and landscape – e.g. National Taiwan Water Resources Institute a good example (landscape architect interview)<br><br>Planning, design and implementation for current issues needs new knowledge: seminars, training programmes, forums to train staff (Parks and Streetlights interview) – especially for sponge city, ecological engineering (Taipei City Land Administration interview)<br><br>Staff recruited through national exam systems, but can also recruit contract-based people with specific skills for specific projects (Taipei City Parks and Streetlights interview)	committee); government-academia research projects to supplement evidence base for decision-making (interview with environmental research institute).
	What processes are there to include different kinds of expertise in decision-making (e.g. social science, humanities,	Collaboration with ICLEI South-East Asian Secretariat on engagement of NGOs and civil society in decision-making for climate change plans in Hanoi (ICLEI-SEAS, 2020)	Brokers/champions (e.g. Classic Design and Planning) contact with local communities to bring research knowledge to local	Public engagement more one-way (e.g. information provision); but also neighbourhood climate change champions to support community-level action

	local/indigenous knowledge)?	Collaboration with communities on piecemeal and project-specific basis when making decisions, e.g. Arts Build Communities projects, community planning of parks by soliciting citizen views (Pham et al., 2013)	communities (landscape architect interview)<br><br>Community university (adult higher education) system – create network of stakeholders in process of doing project and bring local knowledge into the team (landscape architect interview)<br><br>Social science increasingly important and engaged in greenspace activity, especially for insights into issues relating to ageing and open space (landscape architect interview)	(interview with regional environmental NGO)
4. Cross-sector and cross-organisational collaboration	What approaches exist to support cooperation between different departments and sectors? This includes cooperation with other sectors (e.g. private sector, NGOs) as well as different local government departments/	Limited: coordination between government bodies and departments weak, difficult to get people to sit down together (interview with climate change researcher);<br><br>Also problem of developers being able to buy up land and build new developments, regardless of plans? (interview with greenspace researcher; interview with planning consultant)	Long history of community engagement (now on 4th generation), involving not only communities but also stakeholder groups. NGO/community university/private sector partnerships can sustain projects now once professional group has established project and left (landscape architect interview)<br><br>Proactive nature of NGOs and academics, facilitate international learning and	Internally: awareness and recognition of limited cooperation between e.g. Housing Division (Greenspace) and Environment Division (Climate Planning); aiming towards annual forum to facilitate knowledge-sharing (interview with Fukuoka City Green City Promotion Division);<br><br>Externally: expert advisory committees, feeling from participants that advice and opinions are genuinely considered (interview with

			sharing of case studies/ideas (Taipei City Parks and Streetlights interview)<br><br>Co-design of workshops and engagement with NGOs (Taipei City Parks and Streetlights interview)<br><br>Different modes of working: top-down, bottom-up, outside-in (international information), inside-out (collaboration with community universities on projects) (Taipei City Land Administration interview)	academic involved in adaptation planning committee).
	What opportunities are there for public participation in decision-making?	Planning very much top-down, with limited means for citizens to be consulted (interview with greenspace researcher)<br><br>Nonetheless, it is true that social awareness of environmental issues in Hanoi has risen (e.g. tree felling to make way for new mass transit routes) (interview with greenspace researcher)	Consultations with head of neighbourhood; public hearings; opportunity for citizens to write to government to offer opinion (urban planning consultant interview)<br><br>Recognition of importance of involving local people, e.g. work with communities to plan small spaces from bottom up, but at same time consider climate adaptation issues within this (landscape architect interview)<br><br>Important to legislate process of public participation – can	Public engagement more one-way (e.g. information provision, citizen surveys); but also neighbourhood climate change champions to support community-level action (interview with regional environmental NGO).

			formalise engagement. At present, engagement happening within projects, but not part of law (landscape architect interview)<br><br>E-platform to digitalise info, create Garden City Bank, and find information/activists/social groups online (Taipei City Parks and Streetlights interview)	
	How effective are these participatory processes? Can they have a meaningful effect on the outcomes and decisions that are taken?		In neighbourhoods where there is good participation, the neighbourhood gets more information from citizens and hence the quality of the decision can be better (urban planning consultant interview)<br><br>Limited resources for engagement – manage expectations, consensus-based forms of engagement, work with more pragmatic people or opinion-shapers (Taipei City Parks and Streetlights interview)	For techno-scientific expert input yes, to an extent (recommendations not always fully implemented, or mis-interpreted) (interview with academic involved in climate plan committee);<br><br>Public participation in, for example, proliferation of green roofs and gardens, albeit on voluntary basis.
5. Consideration of ethical and justice issues	How equitable is access to key greenspace / adaptation assets within the city?	New residential areas such as Ecopark are very green with lots of trees and lakes – private development, but open areas accessible to general public (interview with urban planning academic). Equally though,	City government concern with inequality – greenspace per capita varies by district, yet several functions not yet covered such as inequality in greenspace function – makes it hard to decide if/where more	Greenspace plans list standards for accessible greenspace, and ratio of green coverage/green area per person for each ward. Area varies considerably between wards – highest green coverage ratio in Sawara/Nishi

		questions over how 'equal' and open this really is (Environmental Justice Atlas, 2015)<br><br>Uneven distribution of both accessible recreational greenspaces (JICA and Hanoi People's Committee 2007; Nguyen 2015) and greenery in general (Nguyen 2018)<br><br>Average recreational greenspace per capita lower in five historical central districts than in four extended inner-city districts. This difference is significant, ranging from 0.25 m^2 per person to 2.58 m^2 per person in ten central districts (Nguyen 2018).	greening is required (landscape architect interview)<br><br>Regulation in urban plan says 10% greenspace, so to meet this criteria remote/derelict areas allegedly included to meet target (Taipei City Parks and Streetlights interview)	Wards and most formal greenspace/person in Nishi/Higashi Wards, least green coverage in Chuo/Hakata Wards and least formal greenspace/person in Jonan/Sawara Wards. (Mabon et al., 2019)
	What data and processes does the city have to understand differences in vulnerability across sections of society?	Vulnerable areas calculated at ward level based on socio-economic data – surveys every 5 years (interview with greenspace researcher);<br><br>But even at government level, knowledge of how to translate this into risk/vulnerability assessment is low (interview with climate change researcher).	For climate change, can derive particularly vulnerable people from economic status (Taipei City Land Administration interview)	Greenspace plan lists elderly, disabled people and children as 'vulnerable' groups (Fukuoka City New Green Basic Plan, 2009)<br><br>Ageing population discussed – on basis of socio-economic data – as issue in greenspace plan, and elderly mentioned in climate plan (Fukuoka City New Green Basic Plan, 2009; Fukuoka City Climate Change Countermeasures Action Plan, 2016)

	Is there explicit consideration of justice and equity issues in municipal greenspace planning with regard to adaptation?	For last 50-60 years, Hanoi has planned for ratio of greenspace under socialist idea that everyone should be equal – but this is only the plan, not reality where real estate and development takes precedence (interview with urban planning academic)	Limits to what can be done with regards to equity, as there is a need to follow a basic plan which is now over 40 years old – now being reviewed, but politically risky to change (urban planning consultant interview)	Historical concern with environmental justice issues in Kyushu (e.g. air and water pollution issues) as motivation for evidence-driven environmental research (interview with Fukuoka Prefecture) – but limited explicit consideration in policy and planning.
	What measures are taken in the city to reduce inequalities and/or benefit the most vulnerable at climate/greenspace interface?	Partnerships/third sector – e.g. Canadian NGO Healthbridge involved in work on parks, around young people (Hanoi Youth and Public Space, 2015)	Open platform for application for urban greening projects, specific preference for preferred communities – can fund NTD50k over 3 years, try to fund all projects but experts can decide which are best and if communities have other resources they may not be funded (Taipei City Urban Development interview)	Greenspace plan lists large-scale greenspace provision for health of children and elderly (Fukuoka City New Green Basic Plan, 2009)

REFERENCES

Baba, K., Matsuura, M., Kudo, T., Watanabe, S., Kawakubo, S., Chujo, A., … Tanaka, M. (2017). *Climate Change Adaptation Strategies of Local Governments in Japan*. https://doi.org/10.1093/ACREFORE/9780190228620.013.597

Environmental Justice Atlas. (2015). EcoPark Satellite City Project, Hanoi, Vietnam | EJAtlas. Retrieved June 12, 2020, from https://ejatlas.org/conflict/ecopark-satellite-city-project-hanoi-vietnam

Fukuoka District Meteorological Observatory. (2017). *Information on climate change predictions for Kyushu and Yamaguchi Prefecture (in Japanese)*. Fukuoka: Fukuoka District Meteorological Observatory.

Hagishima, A. (2018). Green infrastructure and urban sustainability. *AIP Conference Proceedings*, *1931*(1), 020002. https://doi.org/10.1063/1.5024056

Hanoi Youth and Public Space. (2015). HanoiYouthPublicSpace. Retrieved June 15, 2020, from http://www.hanoiyouthpublicspace.com/

Hijioka, Y., Takano, S., Oka, K., Yoshikawa, M., Ichihashi, A., Baba, K., & Ishiwatari, S. (2016). Potential of existing policies of the Tokyo Metropolitan Government for implementing adaptation to climate change. *Regional Environmental Change*, *16*(4), 967–978. https://doi.org/10.1007/s10113-015-0809-y

Ichinose, T., Mikami, T., Niitsu, K., & Okada, N. (2003). *Counteractions for urban heat island in regional autonomies activities in councils of MoE*. https://doi.org/10.11298/taiki1995.37.6_A71

ICLEI-SEAS. (2020). Hanoi – Ambitious City Promises. Retrieved June 15, 2020, from https://acp.iclei.org/city/hanoi/

Jim, C. Y. (2017). Green roof evolution through exemplars: Germinal prototypes to modern variants. *Sustainable Cities and Society*, *35*, 69–82. https://doi.org/10.1016/j.scs.2017.08.001

Leducq, D., & Scarwell, H. J. (2018). The new Hanoi: Opportunities and challenges for future urban development. *Cities*, *72*, 70–81. https://doi.org/10.1016/j.cities.2017.08.003

Mabon, L., Kondo, K., Kanekiyo, H., Hayabuchi, Y., & Yamaguchi, A. (2019). Fukuoka: Adapting to climate change through urban green space and the built environment? *Cities*, *93*, 273–285. https://doi.org/10.1016/j.cities.2019.05.007

Pham, T. L., Nguyen, T. H., Nguyen, T. T., Nguyen, T. H., Phung, M. H., & Le, O. M. (2013). *Improving urban parks in Hanoi*. Hanoi: National University of Civil Engineering.

Taipei City Rain and Waterway Engineering Department. (n.d.). Sponge City. Retrieved June 15, 2020, from https://heo.gov.taipei/cp.aspx?n=F50EC7128A8D18B4

Yoda, H., & Katayama, T. (1998). Climate analysis for urban planning in Fukuoka. *Report of Research Center for Urban Safety and Security*, *Special Re*, 63–78.
